# Supplementary material for: Content-rich biological network constructed by mining PubMed abstracts
Source: BMC Bioinformatics. 2004 Oct 8;5:147. doi: 10.1186/1471-2105-5-147 (PMC528731; doi:10.1186/1471-2105-5-147)
Supplement: Additional File 5 — The original Chilibot query results of the term "long-term potentiation (LTP)" and 22 other terms, limiting the latest references analyzed to the years 1990, 1995, 2000, and 2004. [file 1471-2105-5-147-S5.bz2 › chilibotAdditionalFile5/ltp2000/html/SYNAPTOTAGMIN_SYNAPTOPHYSIN.html]

 


 **SYNAPTOTAGMIN** and **SYNAPTOPHYSIN** 
  
Found 73 abstracts in PubMed,  **30 abstracts were retrieved and analyzed**.  


---

 Search Google  |
 PDF files only 
|  EDU domain only 

---

**Interactive relationship** (e.g. stimulation, inhibition, etc)

- In these investigations we found that the same treatment induced accumulation over 2 fold of other proteins in the secretory pathway, including synaptosomal associated protein of 25 kDa SNAP 25,  **synaptotagmin**  III, synaptobrevin,  **synaptophysin** , and cyclophilin B, and did not affect accumulation of others, including  **synaptotagmin**  I, calnexin, and glucose regulated protein 94.  Ref: 10965922 Endocrinology, 2000
- In subcellular fractionations of MIN6 cells,  **synaptotagmin**  III was recovered in the vesicular fractions containing both insulin and vesicle associated membrane protein 2 VAMP 2, but not in  **synaptophysin**  positive fractions.  Ref: 9392487 Diabetes, 1997

**Parallel relationship** (e.g. studied together, co-existance, homology, etc.)

- In the soma of SN56 cells, the transporter immunoreactivity was similar to that for  **synaptotagmin** , and it colocalized with  **synaptophysin** , but it did not colocalize with SV2.  Ref: 10537046 J Neurochem, 1999
- Variations of  **synaptotagmin**  I,  **synaptotagmin**  IV, and  **synaptophysin**  mRNA levels in rat hippocampus during the estrous cycle.  Ref: 10506530 Exp Neurol, 1999
- The ECL cells displayed immunoreactivity for  **synaptophysin** ,  **synaptotagmin**  III, vesicle associated membrane protein 2 VAMP 2, cysteine string protein CSP, vesicular monoamine transporter 2 VMAT 2, synaptosomal associated protein of 25 kDa SNAP 25, syntaxin, and Munc 18, but not for  **synaptotagmin**  I II and VAMP 1.  Ref: 9369530 Cell Tissue Res, 1997
- SV2B is colocalized with  **synaptophysin**  and  **synaptotagmin** , markers for microvesicles.  Ref: 9648885 J Neurochem, 1998
- The tissues were fixed, cryoprotected, frozen, cut in 15 micrometer sections, and processed immunohistochemically using antibodies against  **synaptophysin** ,  **synaptotagmin**  I, or SNAP 25.  Ref: 10531545 J Comp Neurol, 1999
- Horseradish peroxidase filled vacuoles as wells as vesicles contained the synaptic vesicle membrane proteins VAMP II,  **synaptotagmin** , SV2, and  **synaptophysin** , the vesicle associated proteins rab 3A and synapsin I, and in addition SNAP 25.  Ref: 10579591 Neuroscience, 1999
- In the present study, antibodies to  **synaptophysin** , synapsin, Rab3a and  **synaptotagmin** , and SNAP 25 and syntaxin have been used to learn about the dystrophic neurite equipment of proteins that are necessary for the docking and fusion of synaptic vesicles, and then for exocytosis and neurotransmission.  Ref: 9600213 J Neuropathol Exp Neurol, 1998
- Immunohistochemistry in combination with confocal laser microscopy showed the presence of synaptosomal associated protein of 25 kDa, syntaxin, cysteine string protein, synuclein, synapsin I, synapsin I II,  **synaptotagmin**  I,  **synaptotagmin**  I II,  **synaptophysin** , and synaptobrevin 2 like immunoreactivity in VAChT containing C terminals.  Ref: 10421869 J Comp Neurol, 1999
- We conclude that synaptobrevin,  **synaptophysin** ,  **synaptotagmin** , SNAP 25, and syntaxin are present in gastric ECL cells.  Ref: 9389539 Endocrinology, 1997
- In contrast, no alteration was observed in the levels of the synaptic proteins,  **synaptophysin**  and  **synaptotagmin** .  Ref: 9427500 Brain Res Mol Brain Res, 1997
- The amount of immunoreactivity for  **synaptophysin** ,  **synaptotagmin** , and synaptosomal associated protein 25 did not differ between the young and aged rats.  Ref: 10588583 Neurobiol AgingNeurobiol Aging, 1997
- Granules were labeled for SV2 and  **synaptotagmin**  but not for  **synaptophysin** .  Ref: 9239749 Neurochem Res, 1997
- Other known SNARE and SNARE associated proteins such as vesicle associated membrane protein synaptobrevin 2, SNAP 25,  **synaptophysin** , or  **synaptotagmin**  I could not be immunochemically detected in platelet membrane preparations.  Ref: 9269766 Blood, 1997
- The present results have shown that, although most neuritic senile plaques have chromogranin A and SNAP 25 immunoreactive dystrophic neurites, only a percentage of them contain  **synaptophysin** , and a minority contain  **synaptotagmin**  and Rab3a.  Ref: 9600213 J Neuropathol Exp Neurol, 1998
- Immunoblotting data demonstrated that Rab3a,  **synaptotagmin** , cellubrevin, synaptobrevin 2, syntaxin 1, SNAP 25 and  **synaptophysin**  were well represented in anterior pituitary cells as well as in the corticotroph cell line AtT 20.  Ref: 9472720 Life Sci, 1998
- Varicosities have the potential to be functional sites of transmitter release because they responded to depolarization with calcium influx through voltage gated calcium channels and expressed the synaptic proteins  **synaptotagmin** , SV2,  **synaptophysin** , and a subunit of P Q calcium channels.  Ref: 10537046 J Neurochem, 1999
- The distribution of the three synaptic vesicle proteins SV2,  **synaptophysin**  and  **synaptotagmin** , and of SNAP 25, a component of the docking and fusion complex, was investigated in PC12 cells by immunocytochemistry.  Ref: 9239749 Neurochem Res, 1997
- VAChT containing varicosities in the Renshaw cell area, with a probable origin from motoneuron axon collaterals, exhibited CSP, synapsin I II, and synaptobrevin 1 LI... syntaxin, synuclein, synapsin I,  **synaptotagmin**  I,  **synaptotagmin**  I II,  **synaptophysin**  and synaptobrevin 2 LI.  Ref: 10421869 J Comp Neurol, 1999
- In the CG, two SVP genes,  **synaptotagmin**  I syt I and  **synaptophysin**  II syp II, are coordinately upregulated at the time of target contact.  Ref: 9671670 J Neurosci, 1998
- Membrane proteins of SSV analogues such as  **synaptophysin** , synaptobrevin v SNARE and  **synaptotagmin**  are key proteins of neurosecretion.  Ref: 9474867 Verh Dtsch Ges Pathol, 1997
- Immunoblots of isolated ECL cells demonstrated the presence of SNAP 25, synaptobrevin,  **synaptophysin** ,  **synaptotagmin** , and syntaxin.  Ref: 9389539 Endocrinology, 1997
- Electron lucent vesicles were labeled most intensively for  **synaptophysin**  but also for SV2 and to a lesser extent for  **synaptotagmin** .  Ref: 9239749 Neurochem Res, 1997
- Furthermore the ECL cells display immunoreactivity for vesicular monoamine transporter type 2,  **synaptophysin** ,  **synaptotagmin**  III, vesicle associated membrane protein 2, cysteine string protein, synaptosomal associated protein of 25 kDa, syntaxin and Munc 18.  Ref: 10461354 Yale J Biol Med< MedlineTA>Yale J Biol Med, 1997
- As AD is an aging associated disease, in the present study we examined the developmental and aging related changes in synaptic proteins such as  **synaptophysin** , synaptobrevin,  **synaptotagmin** , synaptosomal associated protein 25 SNAP 25, syntaxin 1 HPC 1 and drebrin in the rat brain.  Ref: 9790966 Biochem Biophys Res Commun, 1998
- Both populations contain proteins that are markers of synaptic vesicles, namely, SV2,  **synaptotagmin** ,  **synaptophysin** , secretory carrier membrane proteins, synaptobrevin, and rab3a.  Ref: 9419350 Proc Natl Acad Sci U S A, 1998
- To investigate the molecular mechanisms involved in synaptic remodeling during the estrous cycle, we analyzed the expression of three presynaptic markers,  **synaptotagmin**  I,  **synaptotagmin**  IV, and  **synaptophysin** , in the female adult rat brain by in situ hybridization.  Ref: 10506530 Exp Neurol, 1999
- There has been a controversy whether these vesicles contain antigens like  **synaptophysin** ,  **synaptotagmin**  and VAMP or synaptobrevin found in high concentration in synaptic vesicles.  Ref: 9239747 Neurochem Res, 1997
